# Revolutionizing Drug Discovery: The Impact of Distinct Designs and Biosensor Integration in Microfluidics-Based Organ-on-a-Chip Technology

**DOI:** 10.3390/bios14090425

**Published:** 2024-09-03

**Authors:** Sheng Yuan, Huipu Yuan, David C. Hay, Huan Hu, Chaochen Wang

**Affiliations:** 1 Centre of Biomedical Systems and Informatics, Zhejiang University-University of Edinburgh Institute (ZJU-UoE Institute), School of Medicine, International Campus, Zhejiang University, Haining 314400, China; 2 Sir Run Run Shaw Hospital, School of Medicine, Zhejiang University, Hangzhou 310020, China; 3 Centre for Regenerative Medicine, Institute for Regeneration and Repair, The University of Edinburgh, Edinburgh EH16 4UU, UK; david.hay@ed.ac.uk; 4 Zhejiang University-University of Illinois Urbana-Champaign Institute (ZJU-UIUC Institute), International Campus, Zhejiang University, Haining 314400, China; 5 Department of Gynecology, The Second Affiliated Hospital, School of Medicine, Zhejiang University, Hangzhou 310020, China

**Keywords:** organ-on-a-chip, drug development, biomedicine

## Abstract

Traditional drug development is a long and expensive process with high rates of failure. This has prompted the pharmaceutical industry to seek more efficient drug development frameworks, driving the emergence of organ-on-a-chip (OOC) based on microfluidic technologies. Unlike traditional animal experiments, OOC systems provide a more accurate simulation of human organ microenvironments and physiological responses, therefore offering a cost-effective and efficient platform for biomedical research, particularly in the development of new medicines. Additionally, OOC systems enable quick and real-time analysis, high-throughput experimentation, and automation. These advantages have shown significant promise in enhancing the drug development process. The success of an OOC system hinges on the integration of specific designs, manufacturing techniques, and biosensors to meet the need for integrated multiparameter datasets. This review focuses on the manufacturing, design, sensing systems, and applications of OOC systems, highlighting their design and sensing capabilities, as well as the technical challenges they currently face.

## 1. Introduction

The process of developing new drugs is a complex, costly, and time-consuming process with a high attrition rate [1]. Specifically, a new drug must undergo laboratory experiments, preclinical studies, and clinical trials (phases I, II, and III) before it can be approved for marketing. Following market entry, it must also undergo phase IV clinical trials and post-marketing approval [2]. Many candidate drugs are eliminated at different stages throughout the development process due to poor efficacy, safety issues, high production costs, and other factors [3]. Consequently, the average development cycle of a new drug takes 10–15 years and costs over 2 billion US dollars [4].

Given these challenges, the pharmaceutical industry has sought more sophisticated drug development models that are better at screening out compounds with serious off-target effects. This urgent need has spurred the development of organ-on-a-chip (OOC) technology based on microfluidics [5]. In OOC systems, microfluidic cell cultures are integrated with circuits that precisely manipulate the cells’ microenvironment to simulate the activities and physiological responses of various human organs, thereby providing an efficient research model for biomedical study [6]. OOC systems have many advantages compared to traditional research methods. They can replicate more complex in vivo environments, including interactions among cells, tissues, and blood vessels. Additionally, OOCs require fewer reagents, cells, and space while facilitating rapid analysis, high-throughput experimentation, and automation [7,8]. Overall, this technology is expected to mitigate the risks and costs associated with new drug development while enhancing efficiency and output quality.

The global demand for OOC technology is rapidly increasing due to its enormous potential in new drug development [5]. It is projected to grow from 131.11 million US dollars in 2024 to 1.3883 billion US dollars by 2032, with a compound annual growth rate of 34.3% during the forecast period [9]. This significant growth trend reflects the market expansion and widespread acceptance and promotion of OOC technology from academic research to commercial applications. As more biotechnology and pharmaceutical companies invest in the research and development of OOC technology, this field is expected to maintain rapid development momentum in the coming years.

This review summarizes the technical basis of OOCs based on microfluidic technology, existing designs, integrated sensors, and their applications in drug discovery and preclinical screening. Additionally, we analyze the main challenges and recent breakthroughs of the technology, highlight future research directions, and discuss the broad application prospects of this technology in drug development, personalized medicine, and disease treatment. Overall, OOC technology is advancing swiftly, especially with respect to enhanced design and the integration of multi-functional sensors. Despite existing challenges, OOC holds significant and expansive potential for the foreseeable future.

## 2. Fabrication and Sensors of Organ-on-a-Chip

### 2.1. OOC Fabrication

**Figure 1:**
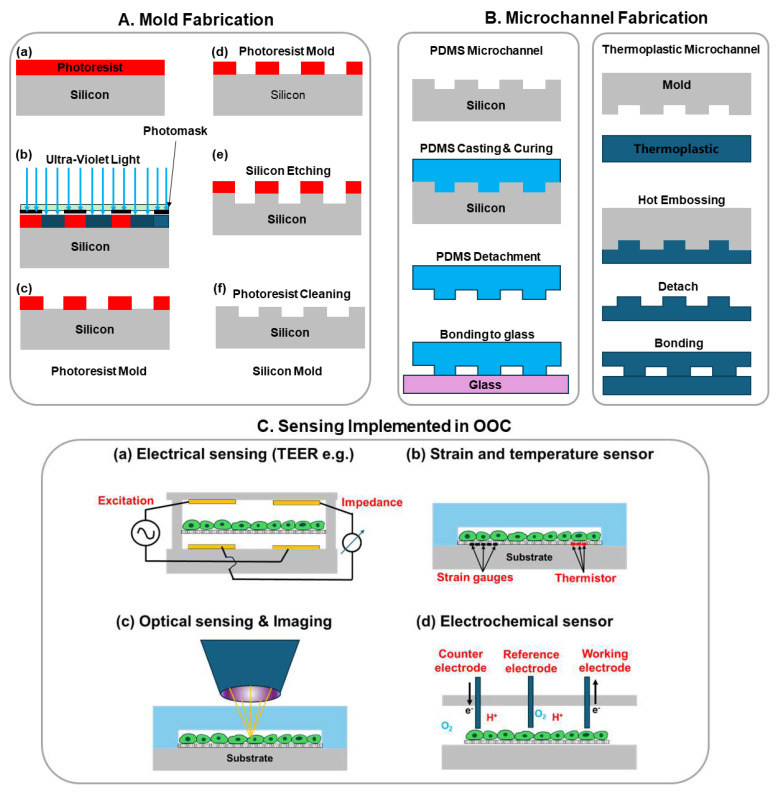
Illustration of OOC chip fabrication: mold fabrication and microchannel fabrication. (**A**) Mold fabrication using conventional UV lithography and etching: (a) Photoresist spincoating and prebake; (b) UV-lithography exposure with a photomask; (c) photoresist development; (d) in cases where photoresist mold is not sufficient, a more reliable silicon mold is fabricated starting with a photoresist mold on silicon as step (c); (e) Reactive ion etching of silicon with photoresist as etching mask; (f) Removing photoresist and rendering a pristine silicon mold; (**B**) Microchannel fabrication using molding for PDMS or hot embossing for thermoplastics; (**C**) Various sensing implemented in OOCs: (a) Electrical sensing mostly impedance sensing such as trans-epithelial electrical resistance (TEER); (b) Strain and temperature sensors mostly made of strain gauges and thermistors; (c) Optical sensing and imaging to investigate the cell morphology and photoluminescence of molecules; (d) Electrochemical sensors detect the presence and concentration of ions, gas molecules, glucose, etc.

The major framework of OOCs is generally based on microfluidic chips, which leverage microfabrication technology or micro-electro-mechanical systems (MEMS) technology to fabricate micrometer-scale channels, reservoirs, valves, etc [10]. The most widely used microfluidic chip technology employs soft lithography [11]. As shown in Figure 1, a mold is first fabricated using ultra-violet (UV) lithography to define microstructures. The typical mold material is photoresist, such as SU-8 (an epoxy-based negative photoresist), or it can be silicon (Figure 1A,B). After the mold is ready, the liquid PDMS is typically mixed with its curing agent in a 10:1 ratio then poured on top of the mold. After curing, the PDMS mold is detached. The final step of the process is to bond the PDMS to a glass cover after plasma treatment. After bonding, the PDMS and the glass substrate can be post-baked for 30 min at 70 degrees C to further improve the strength of the bonding. Microchannels of other materials such as thermoplastic are fabricated using a similar process but with hot embossing as the patterning step instead of material curing. After microchannel patterning, the bonding between thermoplastic can be thermal bonding, adhesive bonding, or ultrasonic bonding [12]. 

Since OOCs normally require fluorescence imaging measurements, even though the PDMS is almost transparent in the visible light range, it emits a certain amount of fluorescence that contributes to the background noise of the fluorescence signal [13]. In addition to glass, which offers much better optical transparency and minimal fluorescence background, researchers have also explored plastic materials such as polystyrene (PS), poly (methyl methacrylate) PMMA, and polycarbonate (PC), which are usually patterned through hot embossing and injection molding [14].

### 2.2. Sensing Systems Implemented in OOC

Sensors can detect and analyze various parameters in biological systems, such as physiological signals, biochemical reactions, and physical changes, providing real-time detection of health or disease status [15]. Within OOC systems, sensors are crucial [16]. Integrated sensors allow for the real-time monitoring and analysis of cell behavior, tissue function, and drug effects, thereby enhancing the OOC system’s capability to simulate and predict biological responses accurately [17]. Figure 1C shows four representative types of sensing including electrical impedance, strain, temperature, and optical and electrochemical sensing. 

Various types of sensors have been developed and integrated into OOCs to measure distinct biological parameters, as illustrated in Figure 1C. For example, Trans-epithelial electrical resistance (TEER) sensors are used to assess the barrier functions of epithelial and endothelial cells in OOC models simulating the intestine, lung, and blood–brain barrier [18,19,20]. Electric cell-substrate impedance sensing (ECIS) sensors provide real-time tracking of cell proliferation, migration, and differentiation, contributing significantly to the study of diverse cell types, including cancer and endothelial cells under various culture conditions [21,22,23].

In addition to these, microelectrode arrays (MEAs) are employed to record the electrical activity of heart and neuronal cells, providing insights into their electrophysiological properties. These sensors have been implemented in heart-on-chip and brain-on-chip models to study the effects of drugs and other interventions [24,25,26]. Strain gauges are used to measure the mechanical forces generated by cells, particularly cardiomyocytes, in heart-on-a-chip models, thereby helping us to understand the impact of drugs and diseases on heart function [24,25].

Environmental factors such as temperature, pH, and oxygen levels are crucial to driving specific cell phenotypes and are monitored using various types of sensors. For instance, a general temperature sensor can monitor temperature in real-time by measuring the electrical resistance change of temperature sensors [26]. Different methods are used for detecting pH and oxygen levels [27]. A microfluidic optical platform constructed by Mousavi Shaegh et al. allows for real-time monitoring of pH and oxygen in OOC systems using low-cost electro-optical devices [24].

Mechanical force, including shear stress, is another key parameter that sensors need to detect. For instance, heartbeat dynamics, a primary indicator for assessing heart health, are studied using various methods [23]. Lind et al. integrated piezoresistive sensors into a multi-layered cantilever beam to guide the growth of cardiac tissue and measure the tissue’s contraction force [25]. In another approach, Aung et al. proposed a detection method that is highly compatible with the OOC design, where the contraction of cardiac tissue generates mechanical forces transmitted to the surrounding hydrogel, resulting in measurable deformation [28].

Electrochemical sensors monitor the metabolic activity of cells by detecting the release of metabolites such as glucose, lactate, and oxygen. For example, amperometric sensors have been used to measure glucose consumption and lactate production in liver-on-chip models [29,30,31]. Optical sensors, particularly those based on photoluminescence, monitor parameters like oxygen levels and pH, providing insights into cellular respiration and metabolic activity. These sensors have been integrated into various OOC models, including liver-on-chip and lung-on-chip, to study cellular responses to hypoxia, drugs, and other stimuli [32,33,34,35]. Sensors for real-time monitoring of specific proteins are also available. For instance, Li et al. designed a novel label-free optofluidic nanosensor for real-time analysis of single-cell cytokine secretion [36]. This sensor monitors the dynamic secretion of cytokines without molecular markers, which can interfere with cell integrity and time resolution.

In most instances, OOC systems incorporate multiple sensor types to simultaneously monitor various parameters. An example of this is a liver-on-a-chip model that combines optical oxygen sensors with electrochemical glucose and lactate sensors. This approach has been used to investigate the metabolic response of liver cells to drugs, offering a more comprehensive understanding of cell function and toxicity [29].

In summary, the integration of diverse sensors into OOC systems underscores the versatility of these platforms and their potential to revolutionize our understanding of cellular behavior and response to stimuli. Examples of sensors implemented in different types of OOC systems are summarized in Table 1.

## 3. Design of Organ-on-a-Chip

**Figure 2:**
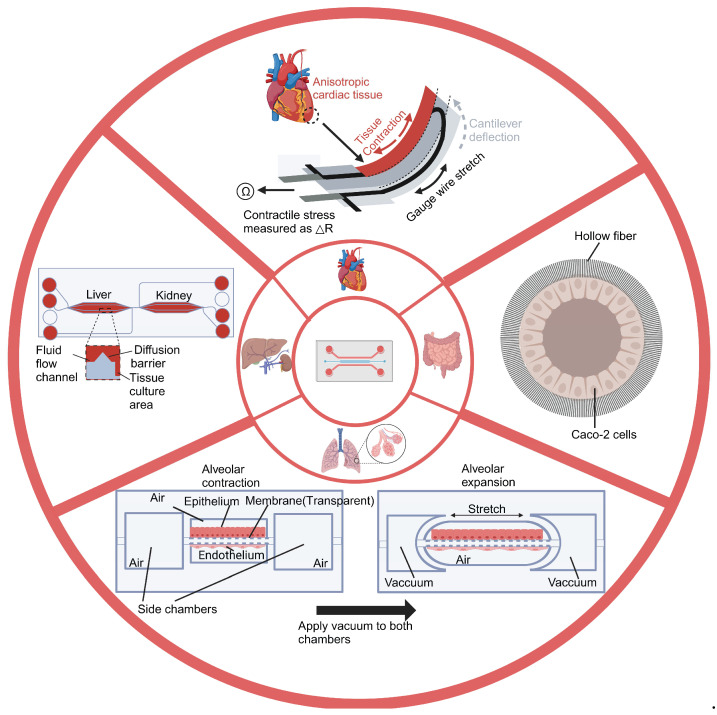
Overview of distinct Organ-on-a-chip designs. The figure shows four chips and their corresponding designs. The lung-on-a-chip simulates the dynamic deformation and gas exchange between the alveoli and capillaries. The heart-on-a-chip simulates the periodic mechanical contraction of heart tissue. The intestinal-on-a-chip simulates the intestinal environment. The liver-kidney chip simulates the interaction between the liver and kidneys in the human body. Created with BioRender.com.

The design of the OOC is crucial to its functionality. The complexity and diversity of these designs determine the breadth and depth of the chip’s application in drug development (Figure 2). In general, microfluidics-based OOCs can be divided into two categories: single-OOCs and multi-OOCs. Single-OOCs simulate the function of a single organ, while multi-OOCs integrate the functions of multiple organs to simulate complex physiological systems.

### 3.1. Single-Organ-on-a-Chip Systems

Single-OOCs are microfluidic devices that mimic the microenvironment and function of a single organ, providing an ideal in vitro model for studying a specific organ’s physiological and pathological processes. Researchers can use these devices to analyze drug interactions with specific organs and assess how organs respond to various physiological conditions [67]. Consequently, these chips are widely used in drug screening, toxicity testing, and disease modeling, identifying key biological mechanisms and thus providing reliable references for clinical trials [5,68]. In this section, we mainly discuss several prevalent types of Single-OOC models and elucidate how their unique designs enhance the in vitro simulation of respective organs. We also briefly summarize the other types of OOCs established in recent studies (Table 2). 

#### 3.1.1. Lung-on-a-Chip 

The lungs are the central organs of the respiratory system in humans and most other vertebrates, playing a crucial role in maintaining cellular respiration by regulating blood oxygen and carbon dioxide levels [81]. Microscopically, gas exchange occurs within millions of alveolar units, the basic functional units of the lung. The alveolar walls comprise a thin epithelial cell layer and a rich capillary network, facilitating efficient oxygen and carbon dioxide exchange [82]. To replicate the biological functions of the lungs effectively, an in vitro lung model must be designed with appropriate cellular components and a structure that supports gas exchange. Microfluidic technology offers precise fluid flow and constant gas exchange, creating a three-dimensional microstructure and microenvironment that mimics the human lung [69,83]. 

**Figure 3:**
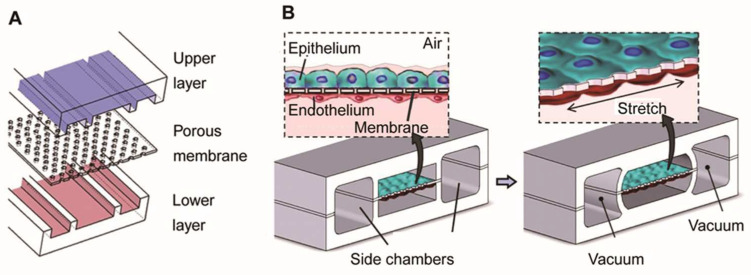
Illustration of the lung model produced by Huh et al. (**A**) The model consists of a PDMS frame and a PDMS porous membrane coated with alveolar epithelial cells in the upper part of the membrane (mimicking air channels) and endothelial cells in the lower part (mimicking microvascular channels). (**B**) The left part simulates the contraction of the alveoli during expiration. The right part simulates the application of vacuum to the lateral lumen, which produces a cyclic stretch that simulates the expansion of the alveoli during inspiration. Reproduced with permission from [69].

Huh et al. produced the first biomimetic microfluidic lung model using classic soft lithography [69] (Figure 3). This model consisted of an upper and lower PDMS (polydimethylsiloxane) frame with a PDMS porous membrane in the middle. The upper and lower PDMS frames were seeded with epithelial and endothelial cells to mimic the microchannels of the airways and blood vessels, respectively. The PDMS membrane, coated with extracellular matrix (ECM) proteins, separated the two chambers to simulate the alveolar–capillary barrier, facilitating gas and nutrient exchange. Applying a vacuum to the two chambers reduces the pressure inside the microchambers, causing the PDMS membrane to deform elastically. This deformation simulates the expansion of the alveoli during inhalation. When the vacuum is released, the PDMS membrane returns to its original state due to its elastic properties, causing the membrane and attached cells to relax, simulating alveolar contraction during exhalation. This elastic recoil effect is crucial for mimicking the dynamic deformation and interaction between alveoli and capillaries. 

In terms of applications, the research team has successfully simulated the inflammatory process in the lungs, including the production of early response cytokines by epithelial cells, the activation of the vascular endothelium, and the adhesion and penetration of white blood cells. Furthermore, the team has explored the pulmonary toxicity of nanoparticles. Findings indicate that respiratory motions amplify the inflammation triggered by nanoparticles, evidenced by heightened ICAM-1 expression in endothelial cells and the increased adhesion and infiltration of neutrophils. The lung-on-a-chip device thus shows great potential for studying the pathological mechanisms of lung diseases, toxicology, and drug development [69,83].

In addition, based on this design, Dasgupta et al. conducted a study on radiation-induced lung injury (RILI), including the effects of radiation on cell structure and function and the potential therapeutic effects of drugs [70]. The study showed that within 6 hours of radiation exposure, both pulmonary epithelial and endothelial cells exhibited DNA damage, increased cell proliferation, upregulation of inflammatory factors, and loss of barrier function; the test drugs (lovastatin and prednisolone) showed potential effects in inhibiting acute RILI. These results support the use of organ-on-a-chip models as a novel method for studying the molecular mechanisms of acute RILI and evaluating new radiation protection therapies.

#### 3.1.2. Heart-on-a-Chip

The heart is the central organ in the circulatory system, primarily responsible for generating pneumatic pressure and driving blood circulation. The myocardium constitutes the main body of the heart. The periodic contraction and relaxation of the uniformly arranged cardiomyocytes in each layer provides the heart’s pumping action [84]. In order to effectively mimic the heart’s biological functions, in vitro heart models must simulate the periodic mechanical contraction of cardiac tissue and allow for real-time monitoring of contraction stress.

**Figure 4:**
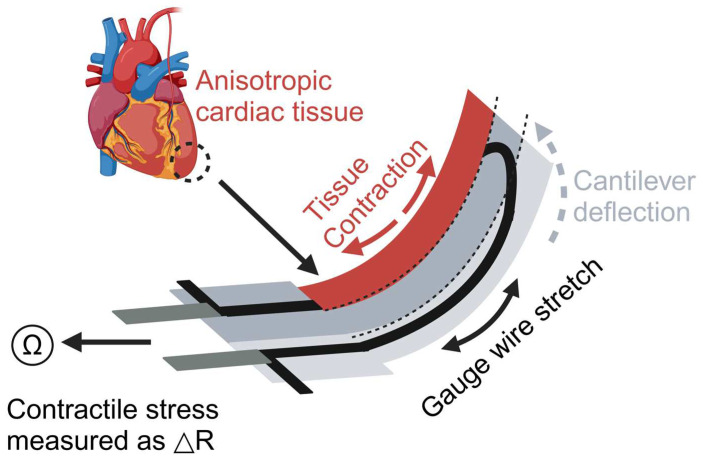
Illustration of the heart model produced by Lind et al. The device is constructed with a U-shaped cantilever that is connected to electrodes for measuring resistance changes. Contraction of cardiac tissue causes deformation of the cantilever, which in turn changes the electrical resistance, and the change in resistance can be used to quantify the contractile stresses exerted on the cardiac tissue. Created with BioRender.com.

Cardiotoxicity is one main reason for drug recalls. Drugs, if cardiotoxicity levels are unidentified in preclinical studies, may cause lethal arrhythmias and death. Lind et al. developed a 3D-printed heart-on-a-chip (HOC) system to study how cardiac tissue responds to drugs [24] (Figure 4). This system was constructed by sequentially printing multiple materials using direct ink writing (DIW). It mainly comprises three layers: a base layer (dextran film, thermoplastic polyurethane (TPU)), a sensor layer (carbon black-doped TPU ink (CB: TPU)), and a tissue-guiding layer (PDMS). These layers provide structural support for the device, placement of sensors, and guidance for cell alignment and assembly. Cardiac muscle cells are seeded onto the tissue guide layer, where they self-assemble into a layered structure that simulates the natural arrangement of cardiac tissue. Furthermore, the integrated sensors can non-invasively detect the contraction stress of the heart tissue and send out the data in real time [85,86]. The researchers conducted a drug dose–response study using this model to investigate the effects of the drug on the contractility and rhythm of the heart tissue. The results showed that a drug (verapamil) produced a negative inotropic effect (attenuated contractility) on cardiac tissue after administration, which is consistent with previous studies. Similarly, a drug (isoprenaline) exhibited a positive inotropic effect (enhanced contractility), which is also consistent with previous studies [87,88]. This model construction method enhances the efficiency and accuracy of heart disease research, allowing for long-term studies of heart tissue function. It provides new tools for heart disease models and drug screening. 

It is worth noting that classic HOC models lack the study of epicardial cells. Bannerman et al. constructed a heart tissue model containing the outer layer of the heart, the epicardium, based on the traditional HOC model containing the inner layer of the heart muscle structure, and tracked the migration of epicardial cells in the experiment [89]. The results showed that under conditions simulating ischemia-reperfusion injury, the epicardial heart tissue had less cell death and less impact on functional characteristics than the control group without epicardial tissue, which laid a foundation for future applications in the study of heart disease and the testing of treatment methods.

#### 3.1.3. Liver-on-a-Chip

The liver, an organ unique to vertebrates, serves as the principal site for critical physiological processes including metabolism, detoxification, bile secretion, immune responses, and the synthesis of various biochemical substances [90]. The structural and functional cornerstone of the liver is the liver lobule, typically hexagonal, comprising millions of hepatocytes. This structure includes hepatocyte plates, hepatic sinusoids, and portal areas, orchestrating the primary physiological functions of the liver through their complex structural and functional integration [91].

**Figure 5:**
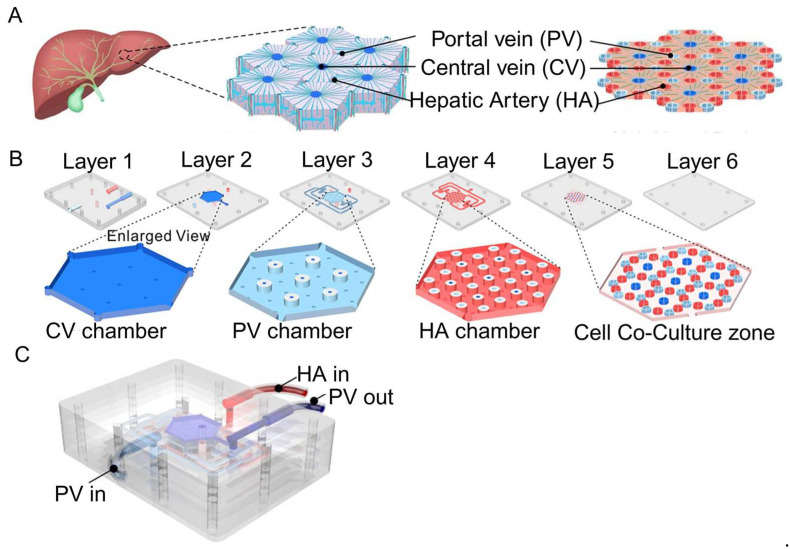
Illustration of the lobules model produced by Ya et al. (**A**) The chip mimics the hexagonal structure of a liver lobule, with the portal vein (PV) at each vertex, the hepatic artery (HA) at the midpoint of each hexagonal edge, and the prominent central vein (CV) at the center of the top surface. (**B**) The chip has a six-layer structure: the first and second layers contain fluid collection chambers for the CV and PV. The third and fourth layers are designed with a raised platform to separate the flow of the PV, HA, and CV. The fifth layer is the co-culture area, which is designed with micro-columns to support cell growth and guide cell alignment to form hepatic sinuses. The sixth layer is the sealing layer, which keeps the entire chip sealed and structurally stable. (**C**) Schematic diagram of the overall structure of the chip and the inlet (PV/HA) and outlet (PV) of the culture medium. Reproduced with permission from [75].

Liver-on-a-chip (LOC) is extensively employed to explore drug metabolism and liver diseases, leveraging designs that emulate the architecture of liver lobules and sinusoids [92]. For instance, lobule chips designed by Ya et al. feature a hexagonal configuration mirroring the liver lobule, incorporating a six-layer assembly with perfusion inlets and outlets at the top, a central liquid distribution system, and a basal cell culture area [75] (Figure 5). The chip simulates the hexagonal structure of the liver lobule, with the portal vein (PV) at each vertex, the hepatic artery (HA) at the midpoint of each hexagonal edge, and the prominent central vein (CV) at the center of the top surface. This design reproduces the blood flow path of the liver lobule, with the cell culture medium entering from the PV and HA inlets and finally converging to the CV through the flow channel system.

The Ya et al. study showed that hepatocytes cultured on a chip exhibited longer-term biochemical functions (far exceeding conventional two-dimensional culture systems), including protein synthesis, urea, and bile acid production. In addition, the researchers also conducted drug toxicity tests and tumor growth simulations. For the toxicity test, the long-term effects of the drug (acetaminophen) on liver function were observed, including changes in enzyme activity and a sustained decrease in cellular metabolism. For the tumor growth simulation, the chip simulated the growth of HepG2 liver cancer cells in liver tissue. In the experiment, HepG2 cells formed tumor spheres on the chip and simulated the tumor microenvironment, including vascular networks and hypoxic areas. 

**Figure 6:**
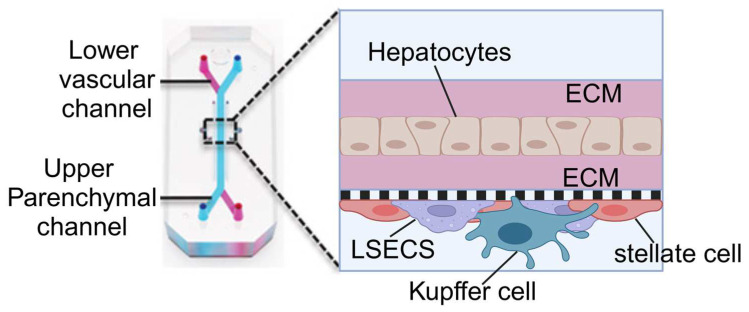
Illustration of the sinus model produced by Jang et al. The chip contains two parallel microchannels, the upper one for hepatocytes and the lower one for blood vessels, separated by a porous membrane. The chip is coated with an extracellular matrix (ECM) to support cell attachment and growth. The upper channel is seeded with primary hepatocytes and the lower channel with liver sinusoidal endothelial cells (LSECs), Kupffer cells (macrophages), and stellate cells. Reproduced with permission from [76].

Similarly, the sinus chip developed by Jang et al., akin to the lung-on-a-chip by Huh et al., includes dual microchannels: an upper hepatocyte-seeded channel and a lower vascular channel lined with sinusoidal endothelial cells [76] (Figure 6). This design may also incorporate Kupffer and stellate cells to stimulate immune responses and fibrotic processes within the liver. The continuous fluid dynamics of the upper and lower channels are controlled by microfluidic channels connected to an external pump system, simulating liver blood flow and enhancing cell–cell interactions and drug metabolism.

Additionally, the research team employed TAK-875, an experimental drug intended for type 2 diabetes management, to investigate the risk of drug-induced liver injury. The results showed that the potential accumulation of TAK-875 and its metabolites in liver cells may lead to cell dysfunction, including inhibition of bile excretion and the activation of cellular stress and inflammatory responses.

Based on the classic LOC, Jiao, Xie, and Xing built a gravity-driven perfusion model of a pump-free LOC [93]. This design avoids the need for an external pump and complex tubing connections, simplifies the construction and operation of experimental equipment, and is more suitable for large-scale drug screening or multi-sample comparison studies. In addition, because there are no external pumps or tubing restrictions, the pump-free chip is easier to integrate with other experimental systems or equipment for a variety of research scenarios. The results found that the chip exhibited similar hepatotoxicity responses to traditional two-dimensional models in drug toxicity testing (aristolochic acid I and its analog aristolochic acid II) but was more sensitive in detecting toxic substances. Overall, this study demonstrates the potential application of this system in drug hepatotoxicity research, especially in high-throughput drug screening.

#### 3.1.4. Other Single-Organ-on-a-Chip Systems

In addition to lung-on-a-chip and heart-on-a-chip, developing other single-OOCs is also crucial for biomedical research and drug development. For example, kidney-on-a-chip (KOC), intestine-on-a-chip (IOC), and spleen-on-a-chip (SOC) are indispensable tools for studying drug metabolism, toxicology assessment, and disease mechanisms.

KOCs replicate the kidney’s filtration, absorption, and secretion functions. These devices simulate the glomerulus or renal tubule and facilitate the study of acute kidney injury, chronic kidney disease, and drug effects on the kidney [94]. Like lung-on-a-chip, glomerulus chips feature an upper channel cultured with glomerular endothelial cells to mimic capillaries and a lower channel cultured with podocytes to replicate Bowman’s capsule. An injection pump adjusts flow rates to simulate physiological and pathological hemodynamic microenvironments [73]. Similarly, renal tubule chips have upper and lower channels cultured with renal tubular epithelial cells to simulate luminal and interstitial regions, respectively, with fluid shear stress applied by an injection pump [74].

IOCs simulate the intestinal environment of the human digestive system and contribute to the study of microbial interaction, intestinal barrier function, and inflammatory bowel diseases [47]. Typically, an IOC comprises intestinal epithelial cells, vascular endothelial cells, and a representative microbial community [47]. In the design by Jalili-Firoozinezhad et al., the upper channel is used to culture intestinal epithelial cells to simulate the intestinal lumen environment, while the lower channel cultures vascular endothelial cells to mimic the vascular environment [71]. Additionally, an anaerobic chamber with an oxygen sensor facilitates microbial culture. 

The SOC replicates the spleen’s function of filtering blood, which helps to study the function of the spleen in blood diseases such as malaria [77]. At the core of the SOC developed by Rigat-Brugarolas et al. are two major microfluidic channels that simulate the closed-fast microcirculation (the fast path through the spleen without filtration, which accounts for about 90% of the total blood flow) and the open-slow microcirculation (the blood passes through the reticular structure in the red pulp of the spleen and is filtered, accounting for about 10% of the total blood flow). To mimic the filtration of blood by the red pulp, the open-slow microcirculation is equipped with a columnar matrix area and micro-contractile structures, which are responsible for increasing the residence time of red blood cells and screening functional red blood cells, respectively.

In addition to the above-mentioned OOCs, which are widely used, more specialized OOCs have been developed recently, including bone marrow-on-a-chip, bone-on-a-chip, blood–brain barrier chips, and lymphoid follicle chips [68,78,79,80]. Bone marrow chips, lymphoid follicle chips, and blood–brain barrier chips all adopt the classic lung-on-a-chip structure, with a porous membrane separating the tissue culture chamber from the blood flow channel [68,79,80]. A micropump continuously perfuses oxygen and nutrients while removing waste products. The blood–brain barrier chip can change the oxygen concentration to induce cells to differentiate into different blood–brain barrier phenotypes [79]. A similar design was used for the bone chips [78]. However, instead of a porous membrane, it uses five PDMS triangular pillars to separate the two vascular channels and the bone microenvironment channel. The bone microenvironment channel can simulate different bone conditions by changing the proportion of three types of bone cells (osteoblasts, osteocytes, and osteoclasts).

### 3.2. Multi-Organ-on-a-Chip Systems

Multi-OOCs integrate various single OOCs to simulate complex interactions between different organs, providing a model that closely replicates physiology [95]. Although still in the developmental stages, significant advancements have been made with systems incorporating two to ten organ models [67]. This section elaborates on two prominent design approaches and their applications in biomedical research.

#### 3.2.1. Horizontal Design

The horizontal design of a multi-OOC involves the integration of multiple organ models that interact functionally with each other. The chambers containing different organs are positioned horizontally and interconnected by microfluidic channels. This configuration is instrumental for studying systemic diseases, multi-organ responses to pharmacological interventions, and the metabolic pathways of drugs in vivo [96]. A typical example is the liver-kidney chip, which combines these two critical organs to assess drug metabolism [97]. The liver, a central metabolic organ, and the kidney, a primary excretory organ, are pivotal in evaluating the pharmacokinetics and potential toxicity of drugs, which are crucial for clinical trial outcomes and market approval [98,99].

**Figure 7:**
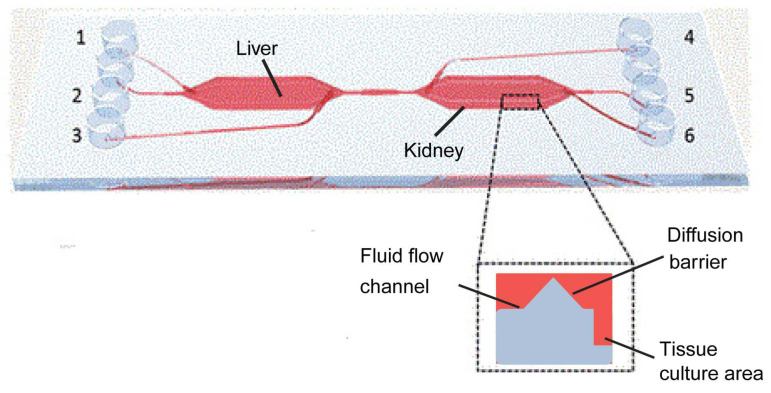
Illustration of the liver-kidney chip produced by Theobald et al. This chip has a total of six inlets/outlets, three on each side (denoted by the number 1–6). And two bioreactors, the liver and kidney, are connected by microfluidic channels that mimic the interaction between the liver and kidney in the human body. Reproduced with permission from [97].

This system is constructed with two interconnected bioreactors, each with independent fluid input and output ports, facilitating isolated or combined organ studies (Figure 7). Each bioreactor comprises fluidic channels, diffusion barriers, and cell culture areas. The fluidic channels ensure precise control of nutrient and waste flow, which is essential for maintaining physiologically relevant conditions. The diffusion barriers minimize convective flow, protecting the cells from shear stress and air bubble formation. The liver and kidney cells are co-cultured in the culture areas under microfluidic conditions simulated by a low-pressure syringe pump system. This setup not only mimics the blood flow-induced shear stress on cells but also enhances the metabolic and absorptive functionalities of the cultured cells, offering insights into cellular responses to various drugs and their metabolites [100].

At the applied level, the system was also used to study the biotransformation and toxicity of aflatoxin B1 (AFB1) and benz[a]pyrene (BαP). In the liver-kidney chip system, the toxins were first exposed to the liver cells, and then their metabolites were transported to the kidney cells via a simulated blood flow. The results showed that this metabolic activity of the liver cells significantly increased the toxic burden on the kidney cells.

The results show that the system can be used to evaluate the toxicity and metabolic response of drugs in a flow-dependent manner, demonstrating the great potential of the horizontally designed multi-OOCs for studying the response of multiple organs to drug intervention and the metabolic pathways of drugs in the body.

#### 3.2.2. Vertical Design

Contrasting with the horizontal arrangement, the vertical design incorporates additional organ models to expand the scope of drug delivery studies and evaluate the effects of different administration routes on drug toxicity and efficacy. Vertical designs are critical in simulating body systems that involve tissue barriers, transcellular transport, and absorption. For instance, integrating skin, intestine, and bone marrow modules facilitates the exploration of transdermal, oral, and intravenous drug deliveries [101].

**Figure 8:**
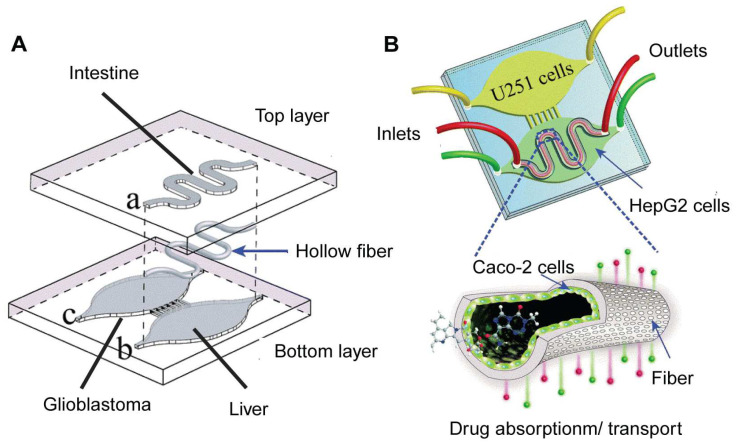
Illustration of the intestinal-liver-cancer chip produced by Jie et al. (**A**) The intestinal bioreactor is embedded in the upper layer (hollow fiber) of the chip; the liver and cancer bioreactors are embedded in the lower layer of the chip. (**B**) The chip has three inlets and outlets for introducing and draining medium or drug solutions, respectively, to maintain the cell growth environment and experimental conditions. U251 cells were cultured in the cancer bioreactor; HepG2 cells were cultured in the liver bioreactor; and Caco-2 cells were cultured in the intestinal bioreactor. Reproduced with permission from [102].

A notable implementation of such a design is the intestinal-liver-cancer chip, developed by Jie et al. [102] (Figure 8). This chip integrates a Caco-2 cell-lined hollow fiber simulating the intestine atop chambers containing HepG2 and U251 cells, representing the liver and glioblastoma, respectively. Drugs administered into the hollow fiber undergo simulated intestinal absorption, subsequent hepatic metabolism, and finally interact with cancer cells, providing a comprehensive model for evaluating the efficacy and toxicity of orally administered glioblastoma therapeutics. The design ensures that all drugs and molecules must undergo cellular processing in the top layer of the intestine chamber before reaching the organ chambers in the bottom layer. This functionality cannot be achieved with a standard horizontal design.

In practical applications, the research team used the model to evaluate the therapeutic effect of a drug combination (irinotecan, temozolomide, and cyclophosphamide) on glioblastoma. The results showed that the drug combination of irinotecan and temozolomide exhibited stronger anti-cancer activity than single drugs and other drug combinations. Interestingly, the authors have demonstrated that metabolites from compounds processed by HepG2 cells were detectable in the extracellular medium, influencing the combined efficacy of the drugs [49]. This provides an alternative to traditional animal models for studying the effect of drug metabolites in cancer therapy. In addition, the multi-OOC model, which employs human cells, might better represent human drug metabolism and efficacy compared to animal models. However, it’s worth noting that the use of an immortalized hepatocyte cell line in the OOC may not fully mimic the body’s drug metabolism in this study. For instance, the metabolite concentrations measured in the current OOC may not be suitable as a reference for further pharmacokinetic studies on the effects of these metabolites. As liver organoid culture techniques mature [75,103], the system could be optimized further by incorporating liver organoids, thereby better replicating the conditions in the human body. Nevertheless, these findings highlight vertical design in multi-OOCs for investigating the impacts of specific or varied drug delivery methods on drug toxicity and effectiveness.

Another implementation of this design is the heart-liver-skin chip, which assesses the kinetics of drug absorption through the skin and its systemic effects [95]. The chip has three bioreactors: skin, heart, and liver. The vertical design of the multi-OOC system simulates transdermal administration (local administration). Cardiomyocytes cultured on microelectrode arrays and MEMS cantilevers within the heart bioreactor at the bottom provide real-time data on mechanical and electrical activities. In addition, this approach mimics body-wide fluid dynamics through a rocking mechanism, ensuring effective medium circulation and simulating physiological interactions among the organs. 

At the application level, the research team used the chip to study the different toxicities of drugs (diclofenac, paracetamol, hydrocortisone, and ketoconazole) between transdermal and systemic administration. The results showed that the effects of all drugs on cardiac and liver function were lower in the transdermal administration mode than in systemic administration. When administered systemically, high concentrations of diclofenac and acetaminophen had a significant effect on cardiac function, such as altering cardiac electrical activity and contractility, while hydrocortisone and ketoconazole mainly affected liver function.

## 4. Applications of OOC in Biomedicine and Clinics

The successful development of OOCs of various organs has significantly benefited biomedical research and clinics. In response to the diverse and complex demands of biomedical research and clinical therapy, a burgeoning body of research has been dedicated to the precise design and development of OOC platforms. These innovative models showcase a paradigm shift in the way biological systems are studied and offer immense potential across various domains. Within the realms of biomedicine and clinical practice, OOC technology holds promise in simulating organ-level functions, disease modeling, drug screening, and personalized medicine approaches. 

Specifically, OOC systems offer a dynamic and physiologically relevant microenvironment that closely mimics the intricate structures and functions of human organs, providing researchers and clinicians with a sophisticated tool for studying disease mechanisms and drug responses. In comparison to traditional methodologies, OOC models offer advantages such as enhanced physiological relevance, scalability, cost-effectiveness, and the ability to perform high-throughput experiments with reduced reliance on animal models. These attributes collectively underscore the transformative impact of OOC technology in advancing biomedical research and clinical applications.

### 4.1. Disease Modeling and Drug Evaluation

Drug discovery is fundamentally an innovative process that relies on an in-depth understanding of biological targets to design or screen potential drug candidates from vast chemical compound libraries [104]. This process is based on extensive research into the pathological mechanisms of specific diseases to identify molecular targets for drug intervention. By utilizing disease-specific OOC systems, researchers can study the pathological mechanisms of diseases. For instance, OOC technology is a critical method for exploring cancer development, including cancer cell phenotype, growth, migration, and invasion [105,106,107,108]. Specifically, Toh et al. created a system that mimics the tumor microenvironment, representing cancer cell migration dynamics in a microfluidic environment and providing an effective platform for evaluating anti-cancer drugs. Similarly, Marturano-Kruik et al. developed a perivascular niche chip for studying breast cancer cell metastasis and drug resistance in bone [105]. Beyond solid tumors, OOCs have also been instrumental in studying hematological cancers [5,109]. A chip-based leukemia model revealed potential mechanisms of chemoresistance in the bone marrow microenvironment of B-cell acute lymphoblastic leukemia (B-ALL), where B-ALL cells utilize factors from the surrounding vasculature, endosteal, and hematopoietic microenvironment (e.g., CXCL12 cytokine signaling, VCAM-1/OPN adhesion signaling, and leukemia-specific NF-κB pathways) to maintain survival and quiescence [5]. These studies suggest that OOCs can potentially replace or complement existing animal models in exploring disease pathomechanisms. One of the primary advantages of OOC models is the utilization of human cells or organoids, or even patient-derived cells or organoids. This approach potentially provides a more accurate reflection of human pathologic mechanisms. However, current OOCs primarily mimic the micro or local environment of targeted organs, encompassing a limited variety of cells or organs. In the human body, disease development and drug responses often involve more complex systemic metabolisms and interactions between organs. For instance, tumor development is not only influenced by the interactions between cancer cells and neighboring fibroblasts, adipocytes, endothelial cells, and macrophages, but also by the infiltration of neutrophils, T cells, and other immune cells from the bloodstream. Additionally, hormones from the endocrine system can also significantly impact tumor progression. These interactions between tumor cells and other cells and organs also play crucial roles in the tumor’s response to drugs. Therefore, there is a need for more complex OOC models that incorporate as many cell types as possible.

### 4.2. Drug Screening and Discovery

Drug screening and development require the identification of targeted and effective compounds from chemical libraries [110]. An efficient and precise screening platform is crucial for successful drug development. Traditional drug screening often relies on the use of common or engineered cell lines. While these approaches offer advantages such as easy access to cell lines and simple platform design, they come with inherent limitations. A major shortcoming is that 2D cultured cells lack complex structure, composition, and intercellular communications found in vivo, leading to differences in drug metabolism and responses. In contrast, OOC technology offers a more sophisticated solution by better mimicking the in vivo environment and addressing these issues. OOC platforms replicate the physiological conditions of organs more accurately, enabling the study of complex cell interactions, tissue responses, and drug effects in a more physiologically relevant manner. By providing a more representative model of human biology, OOC systems have the potential to revolutionize drug screening processes and enhance the efficiency and accuracy of drug development efforts [5]. For example, Jalili-Firoozinezhad et al. investigated radiation-induced cell death and drug responses using a Gut-on-a-Chip platform. By simulating gamma radiation exposure, they observed increases in the generation of reactive oxygen species, cytotoxicity, apoptosis, and DNA fragmentation. Furthermore, pretreatment with the radioprotective drug dimethyloxalylglycine (DMOG) significantly inhibited these damage responses [111]. In addition, Liu et al. developed a vascular-cancer chip model to evaluate the efficacy and impact of drugs on microvascular networks and tumor cells. In this model, human endothelial cells and fibroblasts are co-cultured to form a microvascular network, and colorectal cancer cells are introduced simultaneously. The research team tested the effects of the anti-cancer drugs fluorouracil, vincristine, and sorafenib at different concentrations. The results showed that the effects of these drugs on endothelial cells and tumor cells at different concentrations were dose- and time-dependent [112].

### 4.3. Preclinical Studies

Preclinical studies serve as a critical stage in the drug development process following the initial identification of a drug candidate. The goal is to collect essential feasibility data, conduct iterative testing, and assess the drug’s safety and efficacy prior to its progression to clinical trials [2]. Traditionally, preclinical studies have been primarily conducted using animal studies [113,114]. However, animal studies are inherently low-throughput and cannot accurately predict drug effects in humans due to differences in pharmacodynamic (PD) and/or pharmacokinetic (PK) responses [115,116,117]. In June 2022, the U.S. House of Representatives passed the Food and Drug Amendments of 2022, which included OOC and micro-physiological systems as separate evaluation systems for non-clinical trials of drugs. In August, the FDA approved the first new drug (NCT04658472) to enter clinical trials based solely on preclinical efficacy data obtained from OOC studies.

Different types of drugs require various preclinical studies, including PD, PK, and toxicology testing. PK studies focus on the drug’s concentration in different organ sites during its metabolism, encompassing absorption, distribution, metabolism, and elimination (ADME). PD studies examine the drug’s biological effects on the target organ or tissue and its mechanism of action. Toxicology testing evaluates the potentially harmful effects of the drug to ensure that it does not pose unacceptable risks to humans and the environment. These preclinical indicators can be detected by the OCC system, which offers a powerful tool to optimize preclinical research [118].

#### 4.3.1. PD-PK Testing

Single-OOCs, such as lung-on-a-chip models, play a pivotal role in PK–PD testing for specific organs with unique drug delivery methods. For instance, pulmonary drug delivery is a prominent approach for treating respiratory conditions like asthma and cystic fibrosis [119]. Drugs administered via inhalation traverse bronchial and alveolar tissues, ensuring high bioavailability and therapeutic effects in the lung. In the study by Barros, Costa, and Sarmento, a lung-on-a-chip model was employed to replicate the alveolar–capillary interface, providing a sophisticated platform for predicting PK–PD parameters during drug screening processes. This innovative approach allows researchers to evaluate how drugs interact with lung tissues at the cellular level, offering insights into drug absorption, distribution, metabolism, and excretion within the pulmonary system [120].

Moreover, the advent of multi-organ OOC systems has significantly advanced PK–PD studies by enabling comprehensive investigations under various drug administration conditions [121]. As vital organs for drug metabolism and detoxification, a multi-organ OOC system with liver and kidney chips is particularly important for systematic in situ PK–PD study [122]. Researchers such as Sung, Kam, and Shuler have pioneered the development of multi-OOC systems, including the tumor-liver-bone marrow model, to explore PK–PD relationships [123]. This device comprises multiple cell culture chambers with fluid systems representing different organs. The toxicity and mechanism of action of the anti-cancer drug 5-fluorouracil were evaluated by adding the chemotherapeutic drug and its modulator uracil to the cell culture medium. Herland et al. developed an integrated intestinal-hepatic-renal and bone marrow-hepatic-renal multi-OOC system for PK–PD studies under diverse dosing conditions [113]. This system, utilizing OOC lined with blood vessels to mimic human organ functions, facilitates drug transfer between organs via a microfluidic network containing a blood substitute. By simulating different drug administration routes (e.g., oral and intravenous), researchers were able to accurately predict drug absorption, metabolism, and excretion pathways, in line with clinical data. The intestinal-hepatic-renal chip effectively simulated cisplatin’s metabolism and excretion, aligning with clinical data. The bone marrow-hepatic-renal chip also assessed cisplatin’s pharmacodynamic effects, particularly its toxicity to hematopoietic cells, consistent with clinical observations. This pioneering work highlights the development of OOC platforms in integrating multiple metabolic organs into a single chip, a feature that was lacking in previous studies. On one hand, this system facilitates the discovery and evaluation of the PK and PD parameters of drugs in a systemic metabolic manner, closely mimicking the process in the human body. On the other hand, it potentially enables simultaneous monitoring of therapeutic effects of the metabolites processed by the liver and the potential toxicity of these liver-processed metabolites on the kidney. This multi-organ integration on a single chip offers a more comprehensive and realistic model for studying drug metabolism, efficacy, and toxicity, paving the way for more accurate predictions of drug behavior in the human body.

#### 4.3.2. Toxicology Testing

As noted, preclinical studies traditionally involve laboratory animals. However, many drugs may not exhibit adverse effects in animals during preclinical stages but can cause liver, heart, or kidney damage in patients during clinical trials [124]. This underscores the importance of toxicology testing, where OOCs can serve as more effective and accurate systems than animal models [125]. Ma et al. developed a LOC featuring three-dimensional liver lobular microtissues [126]. These biomimetic microtissues maintain high basal CYP-1A1/2 and UGT enzyme activities, dynamically responding to drug induction and inhibition, effectively simulating toxicology studies in vitro, and providing a screening platform for drug toxicity during combination therapy.

Moreover, multi-OOCs can facilitate the study of drug metabolism between organs. Zhang et al. developed a multi-OOC platform integrating physical, biochemical, and optical sensors for non-invasive and continuous monitoring of microenvironment parameters and dynamic organ responses to drug compounds. The platform assessed drug toxicity in liver and heart chips separately, verifying that anti-cancer drugs like capecitabine exhibit liver toxicity when metabolized by liver cells and cardiotoxicity [110]. In an impressive study by Novak et al., eight vascularized organs, including the intestine, heart, lung, liver, kidney, skin, brain, and blood–brain barrier, were unified [117]. This comprehensive multi-OOC platform allows for systematic toxicity testing across all major organs simultaneously. More importantly, such a platform provides a superior simulation of drug and metabolite circulation within the body. This is crucial because it takes into account potential synergy, feedback, or consequential reactions of different organs to a drug. This kind of holistic approach could revolutionize drug testing, potentially leading to more accurate predictions of drug responses and side effects in humans.

### 4.4. Precision Medicine in Clinics

Precision medicine customizes healthcare choices for individual patients based on their anticipated response or disease risk. Due to genetic and microenvironment heterogeneity, patients often respond differently to drugs, necessitating accurate assessment and optimization of individual efficacy. OOCs can be used to construct patient-specific models for evaluating drug efficacy and safety in specific patient groups, aiding in developing precise treatment plans.

By integrating primary cells from patient donors into an OOC system, it is possible to evaluate the patient-specific drug response [127]. For instance, using epithelial cells from patients with chronic obstructive pulmonary disease allows the OOC system to reproduce the characteristics of the disease [127]. Similarly, by differentiating patient-derived induced pluripotent stem cells (iPSC) into cells that differentiate into blood–brain barrier (BBB) cells and using this as the basis for establishing a BBB chip, researchers can create personalized models that reflect the genetic background and pathological characteristics of specific patients [128].

Despite promising results of cancer immunotherapies in clinical trials, most patients’ responses remain suboptimal, emphasizing the need for precision medicine to optimize treatment [129]. For example, PD-1 checkpoint immunotherapy has shown potential in clinical trials. However, its effectiveness varies due to the genetic heterogeneity of glioblastoma (GBM) in patients and the immunosuppressive tumor microenvironment [130]. Researchers used a GBM-on-a-Chip system to explore immunosuppressive tumor microenvironment heterogeneity and optimized anti-PD-1 immunotherapy for different GBM subtypes, providing a personalized screening approach [131]. In addition, researchers compared CAR T cells from different donors and constructs using micro-patterned tumor arrays (MiTA), finding significant differences in migration, aggregation, and tumor cell killing efficiency, highlighting individual treatment differences [132].

## 5. Technical Challenges and Future Prospects

As OOC and microfluidic technologies rapidly develop, they show significant potential in drug discovery and development but also face numerous technical challenges.

### 5.1. Cost and Manufacturing

First, the high cost of developing and producing OOCs is a primary barrier to their widespread use. Currently, most OOCs are manually fabricated in research laboratories using soft lithography and PDMS, which leads to high costs and design limitations. High-precision microfabrication techniques and expensive materials, such as PDMS and microfluidic devices, are required for precision processing. PDMS also has other disadvantages, such as adsorption for specific drugs and poor light transmission for imaging. Additionally, specialized technicians are needed to maintain and operate these complex instruments. To achieve large-scale applications, it is necessary to standardize the manufacturing process, develop more cost-effective manufacturing methods and materials, and reduce operational complexity and costs. Three possible approaches include current standard manufacturing materials and techniques, advanced additive manufacturing methods, and modular design. For standard manufacturing materials and processes, injection molding and laser cutting can replace PDMS with plastics [97]. Advanced additive manufacturing methods like 3D printing can pre-program and automatically print high-fidelity and controllable tissue structures, complex scaffolds, or device templates [24]. This technology offers a one-step tissue reconstruction and culture platform, potentially revolutionizing OOC manufacturing [133]. Modular design involves breaking OOCs into multiple independent functional modules, which can be flexibly combined to simulate different physiological systems and pathological states. This design enhances system flexibility and applicability while reducing development and production costs.

Integration of sensors also poses a challenge in OOC manufacturing, as they require precise microfabrication processes, including thin-film deposition and photolithography. These processes must be meticulously controlled to achieve the desired sensor geometry and placement within the OOC system. Sensors should be placed in positions that provide the most relevant and least perturbed measurements, which can be influenced by factors like cell distribution and flow dynamics within the device. Optical access is a concern for sensors placed above or below cells, as it may obstruct the view for microscopy-based cell characterization. The use of transparent electrode materials or strategic positioning of the electrodes can help mitigate this issue. Standardization of sensor integration methods and OOC designs is another ongoing challenge. Sterilization of OOC devices is essential for maintaining sterile conditions, but standard sterilization techniques like autoclaving or gamma irradiation can be harmful to some sensor materials. After all, the integration of sensors significantly adds complexity to an OOC system, increasing manufacturing costs and the potential for variability between devices. 

### 5.2. Sensing Systems in OOC

In the development of OOCs, the miniaturization and complexity of the OOC systems necessitate precise and continuous monitoring of various parameters such as oxygen levels, pH, temperature, and metabolite concentrations. However, the integration of real-time sensors also presents a multitude of challenges that span various scientific disciplines. First, material selection is a fundamental aspect of sensor integration. The chosen materials must exhibit biocompatibility and non-toxicity to avoid interference with cell growth or function. Additionally, the materials need to possess the necessary electrical, electrochemical, or optical properties suitable for the various sensing applications simultaneously. Therefore, innovative polymer materials other than PDMS are needed. Second, the design of the sensors is another crucial factor. For electrical sensors like TEER and ECIS, electrode configuration can significantly affect the measurement outcome. The design must ensure uniform electric fields and minimize contact impedance. Third, the long-term stability of the sensors is a critical aspect. Sensors must maintain their performance over the duration of the experiment, which can span several weeks. Issues like sensor drift, degradation of the sensing element, and biofouling, where biological materials adhere to the sensor surface, can affect long-term stability. In addition, the biocompatibility of sensor materials and fabrication processes is paramount to avoid adverse effects on cell viability and function in OOCs. To prevent biofouling, which can alter sensor responses, antifouling coatings may be applied to the sensor surfaces. This is particularly important for sensors that are used over extended periods or for multiple experiments. When multiple sensors are integrated into a single OOC system, there is a risk of crosstalk or interference between the sensors, which can complicate data interpretation. Lastly, the integration of sensors must be compatible with mass production processes, including the use of materials and fabrication techniques that can be scaled up for commercial applications. Addressing these challenges requires a deep understanding of materials science, microfabrication, cell biology, and sensor technology. Advances in these areas are essential for the development of reliable and effective sensor-integrated OOC systems. Future research should focus on overcoming these obstacles to fully exploit the potential of OOC technology in drug discovery and personalized medicine.

### 5.3. Cell Source and Variability

Second, the scarcity and variability of patient-derived cells hinder the development of precision medicine based on OOC systems. OOCs developed from patient-derived materials can play a crucial role in precision medicine. However, the invasive collection of specific samples is impossible in some special conditions. For example, obtaining brain tissue samples from patients with neurological diseases may be too risky and unacceptable. The limited number of patient tissue cells and low proliferation potential can also be problematic. However, iPSCs are cells that have been genetically reprogrammed to an embryonic stem cell-like state, which means they have the potential to differentiate into any cell type in the body [134]. This makes them an excellent source for generating various cell types required in OOC systems. The controlled differentiation of iPSCs could provide a continuous, patient-specific source of cells for OOCs, overcoming the limitations of donor availability and ethical concerns related to primary human cells. 

For instance, iPSCs derived from skin fibroblasts can be an alternative source of unlimited cells to generate autologous target organs or tissues [135,136]. This enables the construction of patient-specific organ OOCs for personalized disease modeling and drug screening [137]. Despite their potential, iPSCs face challenges in OOC systems, including optimizing differentiation efficiency and consistency to ensure stable expression of target cell characteristics. Cells derived from iPSCs may undergo phenotypic drift during long-term culture, necessitating the development of refined culture and differentiation strategies to maintain cell stability [138].

### 5.4. Integration of the Immune System

Third, most OOC systems currently do not integrate immune cells or the immune system. Incorporating the immune system into OOCs represents a significant, yet necessary, frontier in the advancement of this technology. The immune system’s integral role in numerous biological processes and pathologies underscores the current limitations of OOC models that do not include this complex network. The development of an immune system OOC, integrating a diverse array of immune cell types such as T cells, B cells, and macrophages, presents an ambitious research direction. The challenge lies in accurately emulating the intricate interactions between immune cells and other cell types, which encompasses mechanisms like cell signaling and cytotoxicity. The task is further complicated by the organ-specific functionality of the immune system, making the integration of immune system OOCs with existing single-organ or multi-organ OOCs a daunting endeavor. Incorporating a specific sensing system into OOC models to monitor and evaluate immune responses presents another notable challenge. Immune responses, including inflammation, are typically characterized by altered levels of representative cytokines, as well as the proliferation and migration of immune cells. The development and integration of sensors that can detect these changes would significantly enhance the utility and functionality of immune system-integrated OOCs. For instance, electrochemical sensors might be employed to measure cytokine levels, while optical sensors could track cell migration and proliferation [139]. The development of such sensors, however, is not without its challenges. These sensors must exhibit high sensitivity and specificity to accurately detect and quantify the biological markers of interest. Additionally, they must be biocompatible to avoid interfering with the biological processes they aim to monitor. Moreover, they should be capable of continuous, real-time monitoring to track dynamic changes in immune response.

Despite these hurdles, the potential applications are vast. Immune system OOCs could revolutionize drug discovery and clinical trials by facilitating the identification and screening of potential anti-inflammatory or anti-tumor drugs and the evaluation of novel immunomodulatory drugs. Furthermore, when these models utilize cells derived from specific patients, they could enable precision medicine by predicting individual immune responses to treatments, thereby optimizing therapeutic strategies. Despite the challenges, the integration of the immune system into OOC models is pivotal for creating more accurate human biological models, with the potential to drastically enhance our understanding of disease mechanisms and therapeutic development.

### 5.5. Influence of OOC Nanostructures 

Fourth, there is currently no comprehensive study on the influence of OOC nanostructures on the growth and metabolism of organoids. Some studies indicate that nanostructures can significantly affect cell behavior, including adhesion and proliferation. Research shows that moderate surface energy and roughness provide optimal cell adhesion and growth conditions, with higher proliferation and differentiation under these conditions [140]. Additionally, pore nanostructures significantly affect cell behavior. For example, electrospun scaffolds with pore sizes ranging from 50 to 400 microns can influence cell adhesion and proliferation, with specific pore sizes having different optimal effects on various cell types, such as bone marrow mesenchymal stem cells, chondrocytes, and tendon cells [141]. Future research should further explore the impact of different nanostructures on multiple cell types and organoids, providing more theoretical and practical guidance for OOC design and application.

### 5.6. Prospects for the Commercialization of OOC Technology

As technology matures and applications expand, the commercialization of OOC technology will accelerate. Despite the challenges of commercialization, such as limited venture capital in the field, publications in the field of OOC are booming worldwide, and government agencies and universities in developed economies are working with companies to promote the widespread use of OOC technology [142,143,144]. Among them, the EU has adopted Directive 2010/63/EU and established a regulatory framework to support the development of new microphysiological systems and other bioengineering alternatives to animal research, which is of great significance for the promotion of OOC systems [145]. As the use of OOC technology in drug development, disease research, and personalized medicine increases, market demand is expected to grow rapidly, potentially reaching billions of dollars [142]. This growth will drive more investment and research to improve OOCs’ performance and application diversity [142]. As standardized production processes and regulatory frameworks are established, the accessibility and reliability of OOCs will significantly improve, providing more effective and accurate tools for medical research and clinical applications [142]. In the future, OOCs are expected to become a vital pillar of biomedical research, driving innovation and progress in medical technology [144]. 

## 6. Conclusions

This review article focuses on the role that microfluidic-based OOC technologies play in transforming drug discovery. By replicating human physiology, OOCs offer a developing platform for biomedical research, particularly in drug discovery. This technology can potentially reduce the risks and costs associated with drug development while significantly enhancing the efficiency and safety of new drug post-market entry. From early drug discovery to preclinical trials, OOC combined with sensors constitutes a system that can precisely control experimental conditions and deliver real-time data, optimizing the drug development pipeline. Additionally, the various OOC designs, and multi-OOCs, enable the simulation of complex biological processes, organ crosstalk, and pathological states, which is crucial for understanding disease mechanisms and evaluating drug efficacy. Despite the considerable potential of OOC technology in advancing drug development and personalized medicine, it is important to acknowledge the technical challenges and commercialization barriers it faces. Future research must focus on improving manufacturing efficiency, reducing costs, and continually exploring and optimizing OOC designs, as well as developing and integrating novel sensors to support a broader range of biomedical applications. In conclusion, although challenges remain, the future of OOC technology is highly promising. With ongoing technological advancements and enhanced interdisciplinary collaboration, OOC is poised to play a pivotal role in future biomedical research, bringing revolutionary changes in drug development and disease treatment.

## Figures and Tables

**Table 1:** Sensors implemented in different OOC platforms.

OOC Platform	Measurements	Sensors	Applications	Reference
Lung	The barrier integrity of the cells, the secretion of inflammatory markers, Mechanical stress, and changes in cell mechanics	Trans epithelial electrical resistance (TEER) measurement, sodium fluorescein permeability test, ELISA and ATP luminescence assay, and special material that changes color in sync with air pressure	Lung disease models, drug evaluation, mechanical stretching effect	[37,38,39]
Heart	Electrophysiological signals and mechanical contractions of cardiac tissue, and dynamic tissue beating pulse	Microelectrode arrays (MEAs), piezoresistive sensors, calcium transient dye, optical sensing technology, and nanowire probe	Drug evaluation, cardiotoxicity detection	[24,25,40,41]
Liver	Oxygen concentration, cell growth population	Electrochemical dissolved oxygen sensors produced by inkjet printing technology, electrochemical impedance spectroscopy, and amperometric sensors	Metabolic activity monitoring, hepatotoxicity tests	[29,30,31,42]
Intestine	pH, oxygen, temperature, barrier integrity, ion flow resistance, sequential impedance measurement and cell migration	Fluorescent probes, TEER sensors, electrochemical sensors, electrical cell-impedance sensors, monitoring sensors	Barrier function test, ion transport monitoring, anti-inflammation test, human disease models	[43,44,45,46,47,48,49,50,51]
Brain	pH, oxygen, temperature, shear stress, secreted molecules (e.g., cytokines, insulin), blood flow, cell viability, cell-cell interactions, and BBB crossing of drugs and nanoparticles	MEA, External sensor-integrated BOC (TEER measurement and multi-parameter measurement), and internal sensor-integrated BOC (microelectrode arrays and multi-sensor integration platform)	Real-time brain activity monitoring, neurodegenerative disease model, drug development and screening, pre-clinical test of novel therapies	[32,52,53,54,55,56,57,58,59,60,61]
Skin	pH, oxygen, temperature, tight junction formation	Optical pH, oxygen and temperature monitors, TEER sensors, electrochemically activated immune biosensors attached to physical microelectrodes	Skin barrier function test, drug evaluation, toxicity test, biomimetic artificial skin model	[62,63,64,65,66]

**Table 2:** Designs of different Single- Organ-on-a-Chips.

Organ Type	Special Structure	Morphological Simulation	Environmental Simulation	Special Indicator Tests	General Indicator Tests	References
Lung	Alveoli	Dynamic deformation and gas exchange between alveoli and capillaries	Simulating the gas exchange environment during respiration	Gas exchange efficiency	Temperature, pH, oxygen concentration, cell viability, etc.	[69,70]
Heart	Myocardial tissue	Periodic mechanical contraction of heart tissue	Simulating the electrophysiological environment and mechanical stress during heartbeats	Contraction stress of heart tissue, electrophysiological parameters		[24]
Intestine	Intestinal epithelial cells	Periodic mechanical contraction of the intestine; interaction among intestinal epithelial cells, vascular endothelial cells, and microbiome	Simulating the chemical environment inside the intestine, including pH and microbial communities	Barrier function, microbiome balance, inflammation markers		[71,72]
Kidney	Glomerulus	Imitates the filtering action of the kidney glomerulus	Simulating fluid flow, electrolyte concentration gradient, and pressure changes	Glomerular filtration rate, metabolite concentration, renal tubule reabsorption function		[73,74]
	Renal tubules	Imitating the reabsorption function of the nephron				
Liver	Liver lobule	Imitating the special shape of the liver lobules and the multiple blood vessels through the liver lobules	Simulating the liver’s metabolic environment, including oxygen concentration, nutrient, and metabolite concentrations	Metabolic activity, toxicity response, liver enzyme activity		[75,76]
	Hepatic Sinus	Cultivation of endothelial cells from perforated, discontinuous hepatic sinusoids and associated macrophages				
Spleen	Spleen red pulp	Imitates the red bone marrow, stores red blood cells and white blood cells, and screens for healthy red blood cells	Simulating the closed-fast and open-slow microcirculation in the spleen	Mechanical and physiological responses of red blood cells		[77]
Bone	Bone marrow	Three-dimensional bone tissue and bone marrow cavities to mimic the spatial layout of bone	The hematopoietic microenvironment includes stromal cells that support hematopoietic stem cells, vascular networks, signaling molecules and cytokines that regulate cell activity, marrow signaling molecules, and cytokines that regulate cell activity.	Hematopoietic function, cell type, cytokine level		[68]
	Osteoblasts, osteocytes, and osteoclasts	By adjusting the ratio of osteoblasts, osteocytes, and osteoclasts, different bone conditions can be simulated.	The permeability of the vascular system under different bone conditions is simulated through a simulated vascular channel lined with endothelial cells.	Cell co-culture ratio, vascular permeability, tissue mineralization level		[78]
Brain	Blood–brain barrier	Cultured human brain microvascular endothelial cells, human brain astrocytes, and pericytes formed a blood–brain barrier	Simulates the hypoxic microenvironment with less oxygen that the blood–brain barrier is exposed to during development.	TEER, apparent permeability, tight junction protein expression, efflux pump function,		[79]
Lymphatic system	Lymphoid follicle	Using B and T cells, ectopic lymphoid follicles were simulated in 3D extracellular matrix gel	3D extracellular matrix gel as a platform for the spontaneous assembly of ectopic lymphoid follicles.	Lymphoid follicle formation and number, B cell activation status, cytokine secretion		[80]

## Data Availability

Not applicable.
